# Distribution of triclosan-resistant genes in major pathogenic microorganisms revealed by metagenome and genome-wide analysis

**DOI:** 10.1371/journal.pone.0192277

**Published:** 2018-02-08

**Authors:** Raees Khan, Nazish Roy, Kihyuck Choi, Seon-Woo Lee

**Affiliations:** Department of Applied Bioscience, Dong-A University, Busan, Republic of Korea; Monash University, AUSTRALIA

## Abstract

The substantial use of triclosan (TCS) has been aimed to kill pathogenic bacteria, but TCS resistance seems to be prevalent in microbial species and limited knowledge exists about TCS resistance determinants in a majority of pathogenic bacteria. We aimed to evaluate the distribution of TCS resistance determinants in major pathogenic bacteria (N = 231) and to assess the enrichment of potentially pathogenic genera in TCS contaminated environments. A TCS-resistant gene (TRG) database was constructed and experimentally validated to predict TCS resistance in major pathogenic bacteria. Genome-wide *in silico* analysis was performed to define the distribution of TCS-resistant determinants in major pathogens. Microbiome analysis of TCS contaminated soil samples was also performed to investigate the abundance of TCS-resistant pathogens. We experimentally confirmed that TCS resistance could be accurately predicted using genome-wide *in silico* analysis against TRG database. Predicted TCS resistant phenotypes were observed in all of the tested bacterial strains (N = 17), and heterologous expression of selected TCS resistant genes from those strains conferred expected levels of TCS resistance in an alternative host *Escherichia coli*. Moreover, genome-wide analysis revealed that potential TCS resistance determinants were abundant among the majority of human-associated pathogens (79%) and soil-borne plant pathogenic bacteria (98%). These included a variety of enoyl-acyl carrier protein reductase (ENRs) homologues, AcrB efflux pumps, and ENR substitutions. FabI ENR, which is the only known effective target for TCS, was either co-localized with other TCS resistance determinants or had TCS resistance-associated substitutions. Furthermore, microbiome analysis revealed that pathogenic genera with intrinsic TCS-resistant determinants exist in TCS contaminated environments. We conclude that TCS may not be as effective against the majority of bacterial pathogens as previously presumed. Further, the excessive use of this biocide in natural environments may selectively enrich for not only TCS-resistant bacterial pathogens, but possibly for additional resistance to multiple antibiotics.

## Introduction

The surge of pathogenic bacteria resistant to antimicrobials is a major concern for global public health [1], which calls for a need to develop effective antibiotics [2]. Previous reports suggest that microbial resistance to antimicrobial compounds is directly correlated with biocide and antimicrobial use [3].

The biocide triclosan [5-chloro-2-(2,4-dichlorophenoxy)phenol] (TCS) is widely used in a variety of personal care products [4–6]. TCS blocks bacterial fatty acid biosynthesis by targeting the highly conserved enoyl-acyl carrier protein (ACP) reductase (ENR) [7]. However, various mechanisms are known that confer TCS resistance in bacteria, namely (i) ENR overexpression [8]; (ii) the presence of mutated and/or TCS tolerant ENR [9]; (iii) modulation of the outer membrane [10]; and (iv) upregulation of efflux pumps [8, 11]. In addition, studies have found that TCS exerts selective pressure and induces co- or cross-resistance to other antibiotics [9, 12–16]. The mechanisms that underlie TCS-associated co- or cross-resistance are either unknown or attributed to different co-localized antibiotic resistance genes (ARGs) and/or efflux pumps [17]. Excessive use of this biocide has resulted in various environmental and human health concerns [4–5]. In September 2016, U.S. Food and Drug Administration banned over-the-counter antiseptic wash products containing TCS and 18 other antimicrobial agents based on the safety concerns of their long-term use and insufficient evidence demonstrating protection against pathogenic organisms to reduce the spread of illnesses and infections [18]. Other countries, including the members of European Union, have banned or restricted use of this biocide in certain consumer products [19]. In addition, recent reports suggest that TCS is a potential endocrine disrupter [20], inhibitor of various important enzymes of the human body [21–22], elicitor of skin and pulmonary allergies [23–24] and occupational asthma [25]. TCS can potentially alter the microbiome of newborns [26] and was reported to enrich the gut bacterial genes related to TCS and other antibiotic resistance [27].

The majority of enzymes involved in type II fatty acid synthesis are relatively conserved among bacteria [28] with the exception of ENR, which catalyzes the final enoyl reduction step of the fatty acid elongation cycle. To date, four ENR isozymes have been reported from bacteria, FabI [29], FabL [30], FabV [31], and FabK [32]. All ENR isozymes are members of the short-chain dehydrogenase reductase (SDR) superfamily with the exception of FabK [33]. Despite sharing minimal sequence identity (15–30%), these ENRs not only have significantly conserved structures, but also a largely conserved folding pattern. These characteristics allow specific sequence motifs to be assigned, the most important of which are for coenzyme binding and the enzyme active site [34–36]. FabI is the only effective target ENR for TCS, however, substitutions at key amino acids in FabI can confer the organisms either resistant or refractory to TCS. Natural or induced mutations in FabI associated with TCS resistance include G93V, G93S, G93A, M159T, F203L, F203C, F203A, and S241F [17, 37–39]. Other ENRs are either partially TCS resistant (FabL) or completely tolerant to TCS, such as FabV [31], 7-αHSDH, and FabG-like ENR homologues [17] whereas only FabK ENR has shown either moderate resistance [17] or complete tolerance [32]. ENRs have been potential targets for the development of new antibiotics for decades, and a variety of synthetic ENR inhibitors have been developed or are under development [40].

For over 40 years, TCS has been globally incorporated into a variety of consumer products [5] under the premise that it confers protection against pathogenic bacteria. However, studies evaluating the effectiveness of TCS have been limited to examining a few pathogenic microorganisms. Most studies on TCS effectiveness/resistance were performed using either laboratory grown *Escherichia coli* or a few other human pathogens, the majority of which were resistant to TCS because of the presence of TCS resistance determinants in their genomes. However, our understanding of TCS resistance and TCS resistance determinants among the majority of other human pathogens and soil-borne plant pathogens, which constitute pools of potentially transferrable resistance determinants, is limited. Considering the excessive ongoing use of this biocide, there is a dire need to determine whether the majority of human pathogenic bacteria are resistant to TCS.

*In silico* genome-wide studies have been widely used to uncover various aspects of the genome, such as antimicrobial resistance [41–43] and antimicrobial targets [44–45]. In this study, we used *in silico* genome analysis of the 183 FabI carrying most common human pathogens [46, 17] and 48 soil-borne plant pathogenic bacteria [17] to investigate the distribution of genes that may confer TCS resistance, which is a potential threat for human and plant health with the continued use of TCS. Further, we extends our study to evaluate the abundance of potential pathogenic genera in TCS contaminated environments. To our knowledge, this is the first study investigating TCS resistance determinants in the most common human pathogens and soil-borne plant pathogens.

## Methods

### Bacterial strains, culture conditions, and DNA isolation

The bacterial strains used in this study were listed in S1 Table and Table 1. These bacterial strains were routinely grown at their optimal growth temperature (refer to S1 Table) and in their optimal growth media (agar or broth) supplemented with appropriate antibiotics. The antibiotic concentrations used were as follows; TCS, 0.5–600 μg/ml and ampicillin 100 μg/ml. Genomic DNA was isolated from selected strains using Dokdo-Prep^TM^ Bacterial Genomic DNA Purification Kit (ELPIS BIOTECH) according to the manufacturer’s protocol. pGEM-T Easy (Promega) vector was used for further subcloning of representative TCS resistant determinants in *E*. *coli* DH5α. Recombinant plasmid DNA was isolated using FavorPrep plasmid extraction mini kit (Favorgen Biotech Corp).

**Table 1:** Evaluation and comparison of the observed phenotype with predicted genotype for TCS resistance.

Strain	Potential TCSRD	Genotype	Observed phenotype	MIC* (μg/ml)
*Aeromonas salmonicida* subsp. s*almonicida* CIP 103209	*FabV*(2), *AcrB*	CTT	CTT	600*
*Bacillus subtilis* subsp. *subtilis* 168	*FabIm*, *FabL*	MODR	MODR	30
*Bacillus subtilis* subsp. *subtilis* JH642	*FabIm*, *FabL*	MODR	MODR	30
*Bacillus velezensis* G341	*FabIm*, *FabL*^**€**^	MODR	MODR	135
*Burkholderia pyrrocinia* CH-67	*FabIm*, *FabV*, *FabK*^**€**^, *AcrB*	CTT	CTT	600*
*Chromobacterium violaceum* ATCC 31532	*FabIs*, *FabK*, *AcrB*	CTT/MODR	CTT	600*
*Escherichia coli* DH5a	*FabIs*, *AcrB*	LR/SUS	LR/SUS	1
*Escherichia coli* BL21(DE3)	*FabIs*, *AcrB*	LR/SUS	LR/SUS	1
*Escherichia coli* DH10B	*FabIs*, *AcrB*	LR/SUS	LR/SUS	1
*Escherichia coli* MG1655	*FabIs*^**€**^, *AcrB*	LR/SUS	LR/SUS	1
*Pectobacterium carotovorum* subsp. *carotovorum* PCC21	*FabIm*, *AcrB*	MODR	MODR	115
*Pseudomonas fluorescens* 2–79	*FabV*, *AcrB*	CTT	CTT	600*
*Pseudomonas putida* KT2440	*FabV*, *FabK*, *AcrB*	CTT	CTT	600*
*Pseudomonas syringae* pv. *tomato* DC3000	*FabIs*, *FabK*, *AcrB*	CTT/MODR	MODR	75
*Ralstonia solanacearum* GMI1000	*FabIs*, *AcrB*^**€**^	LR/SUS	LR/SUS	1
*Ralstonia solanacearum* K60-1	*FabIs*, *AcrB*	LR/SUS	LR/SUS	1
*Xanthomonas oryzae* pv. *oryzae* KACC 10331	*FabV*(2)^**€**^, *AcrB*	CTT	CTT	600*

**Symbols and abbreviations:** FabIs, TCS sensitive FabI without previously known TCS resistance associated substitution(s); FabIm, TCS resistant FabI with previously known TCS resistance associated substitution(s); LR/SUS, low resistance/susceptibility (MIC in the range of 0.5–2 μg/ml); MODR, Moderate resistance (MIC in the range of 10–350 μg/ml); CTT, completely triclosan tolerant (MIC in the range of ≥600 μg/ml); CTT/MODR, completely triclosan tolerant or moderate resistance; TCSRD, Triclosan resistance determinants

*, The levels of TCS resistance of all bacterial strains were determined up to the maximum level of 600μg/ml TCS

€, Selected TCSRD for *In Vivo* TCS resistance test in *E*. *coli* DH5α.

### General DNA manipulations

Standard recombinant DNA techniques were carried out as described previously [47]. Primers used in this study were synthesized commercially at the DNA sequencing facility of MacroGen (Seoul, Korea). Nucleotide and amino acids sequences of the selected pathogenic and non-pathogenic bacterial genomes and their TCS resistance determinants were analyzed using the BLAST and ORF finder online services provided by the National Center for Biotechnology Information (NCBI) [48]. Multiple alignment analysis was performed using BioEdit software in combination with GeneDoc, DNA club and Genome Compiler.

### TCS resistance and determination of minimum inhibitory concentration (MIC)

To test if TCS resistance of bacterial pathogen can be inferred from the presence of putative TCS resistance genes, 17 different laboratory strains were selected for which the whole genome sequence (WGS) information was available (S1 Table). Comparative genomic analysis and search for TCS resistance determinants in these organisms were carried out using TCS-resistant gene (TRG) database (see below for details). In summary, to identify TRG sequence reads, a similarity search was performed between individual human-associated pathogenic bacteria or soil-borne plant pathogen genomes (subject sequences) and the TRG reference database (query sequences) using NCBI BLASTp analysis. Annotated sequence reads were selected that had ≥ 27% amino acid sequence identity with the query sequence and were further analyzed. According to the presence, absence or various combinations of TRG homologues, the bacterial strains were classified into various categories of TCS resistance genotypes and predictable phenotypes, such as low resistance/susceptibility, moderate resistance and complete TCS tolerance. These bacterial strains were examined for TCS resistance in their corresponding growth media with various concentrations of TCS (see details below). TCS resistance of the bacterial strains was compared with predicted genotype of the corresponding bacteria.

The MIC of TCS for the selected bacterial strains was determined in a similar way as previously described [17]. Briefly, bacterial cells were first grown to an OD_600_ of 1.0, and the bacterial suspensions were further serially diluted 1×10^5^ colony-forming units (CFU)/ml. These cell suspensions (1×10^5^ CFU/ml) were spreaded onto corresponding growth media containing TCS in the range of 0.5–600 μg/ml. The culture plates were incubated at optimal growth temperature (S1 Table) for 3 days to one week depending on the growth pattern of the bacterial strains. This experiment was carried out in triplicates for various TCS concentrations. TCS resistance profiling data for all tested bacterial strains in this study were deposited in the National Center for Biotechnology Information (NCBI), under BioProject PRJNA387628.

### Subcloning of TCS resistance determinants

To validate whether the predicted potential TCS resistance determinants confer resistance to TCS, candidate TCS resistance determinants including FabL, FabK, FabI, AcrB and FabV from selected bacterial strains were cloned and investigated for TCS resistance in *E*. *coli* DH5α (Table 1, S2 Table). All of the five selected genes (along with their corresponding Shine-Dalgarno sequence) were amplified from genomic DNA of bacterial strains using gene-specific primers (S2 Table). PCR amplification was performed as follows: an initial denaturation step at 95°C for 5 min; 30 cycles of denaturation at 95°C for 30 s, annealing at the specified temperature (S2 Table) for 30 s, and extension at 72°C for 1 min; and a final extension step at 72°C for 5 min. The amplified PCR products were subsequently cloned into pGEM-T Easy vector (S3 Table). Recombinant pGEM-T Easy plasmids were introduced into *E*. *coli* DH5α. TCS resistance of *E*. *coli* DH5α carrying the recombinant plasmid were investigated on LB agar supplemented with various concentrations of TCS (0.5–600 μg/ml). A negative and positive control *E*. *coli* DH5α carrying either pGEM-T Easy alone or a metagenomic TCS resistant 7-AHSDH like ENR in pGEM-T Easy, respectively were also included in the experiment.

### Selection of human-associated pathogens and soil-borne plant pathogenic bacteria

To identify TCS resistance determinants, *in silico* analysis was performed on selected human pathogenic bacterial strains (N = 183) and soil-borne plant pathogenic bacteria (N = 48). Many of the human pathogens selected are well-known pathogens that have been previously listed [17, 46]. These common human-associated pathogenic and few non-pathogenic bacteria include bacteria of the oral cavity, skin-associated bacteria, food and water-borne bacterial pathogens, zoonotic bacterial pathogens, nosocomial bacteria, emerging pathogens, and some beneficial resident flora (S4 Table). Along with reports of TCS accumulation from wastewater, wastewater treatment plants, sewage sludge [49–50] and sediments [51], in some Asian countries such as Vietnam, the use of sediment as fertilizer was also reported [52]. Therefore, we hypothesized that the sewage sludge from wastewater treatment plants and sediment containing TCS may selectively enrich soil-borne plant pathogens as pathogens may carry TCS resistance determinants. Hence, we extended our *in silico* study to include 48 soil-borne plant pathogens (S5 Table) that cause serious plant diseases across many species, to examine whether they contain TCS resistance determinants.

### Construction of a TCS-resistant gene (TRG) database for similarity search

A previously published TRG database [17] was used in this study for similarity search, [53] (Supplementary Data 2, sheet 2 named as TRG-Reference database of the mentioned paper). However, this database was slightly modified to contain the deduced full-length amino acid sequences of well known prototypic and metagenomic ENRs and the AcrB efflux pump subunit (S6 Table). The prototypic ENRs of the TRG database included: (i) TCS-sensitive FabI from *E*. *coli* [54], (ii) mildly TCS-resistant FabL from *Bacillus subtilis* [30], (iii) TCS-tolerant FabV from *Vibrio cholera* [31], (iv) TCS-refractory FabK from *Streptococcus pneumonia* [32], (v) TCS-tolerant metagenome-derived 7-αHSDH ENR [17], and (iv) multidrug efflux pump subunit AcrB from *E*. *coli* [11]. The *AcrB* efflux pump subunit of the TRG database shared significant identity with the well-known TCS resistant efflux pump protein homologues [55] retrieved from BacMet [56] and NCBI databases (S7 Table). This indicates that the AcrB efflux pump subunit of TRG database is a good candidate for genome wide searches of similar efflux pump homologues which may confer resistance to TCS.

### Comparative search for TCS resistance determinants using a TRG database

TRG sequence reads were identified by performing a similarity search between individual human-associated pathogenic bacteria or soil-borne plant pathogen genomes (subject sequences) and the TRG reference database (query sequences) using NCBI BLASTp analysis. Annotated sequence reads were selected that had ≥ 27% amino acid sequence identity with the query sequence [34–36]. Protein homologues, which were homologous to the proteins in the TRG database, were selected for further comparative analysis, while other homologues, which were similar to hypothetical proteins, were not included in further analysis. Since TCS is purposely used against human pathogens, and FabI is the only known effective target of TCS, human pathogens were further analyzed *in silico* based on either the presence of FabI alone or with other TCS-resistant determinants. Human pathogens that lacked FabI ENR were excluded from this study. However, no such criteria were applied in the analysis of soil-borne plant-associated pathogens. FabI homologues in these organisms underwent an additional search for previously known TCS resistance-associated substitutions such as G93V, G93S, G93A, M159T, F203L, F203C, F203A, and S241F [17, 37–39].

### Sample collection for microbiome analysis

Previously collected soil samples dated 19^th^ August 2009 from alluvial soil (AS) and industrially contaminated soil (ICS) were stored in sterile zipper bags at -80^°^C and processed for DNA extraction and subsequent MiSeq sequence analysis [17]. ICS samples were collected from the Gam-geon stream (Sasang-Gu, Busan, Republic of Korea), which is a highly contaminated stream receiving the combined sewer effluent from many industries, and is in an area that has been highly urbanized by a number of industries, including machine accessories manufacturers, chemical plants, cosmetics, plywood and lumber processing among others, since 1968 [17]. AS samples were collected from Eulsukdo Island, which is a unique ecosystem where the Gam-geon stream joins the Nakdong River and converges into the East Sea, which is a marginal sea of the Pacific Ocean. Each soil sample was processed in duplicate (two technical replicates for each soil type), and both AS and ICS samples were tested previously to be TCS contaminated where TCS was detected at approximately 0.66–1.29μg/L in these samples [17].

### DNA extraction and MiSeq sequence analysis

Soil samples were homogenized and metagenomic DNA isolation was performed using the Fast DNA^TM^ SPIN kit for soil (MP Biomedicals, USA) according to the manufacturer’s protocol. Extracted DNA samples were quantified using a NanoDrop 2000 spectrophotometer (Thermo Fisher Scientific, Wilmington, DE, USA). PCR amplification of the 16S rRNA gene was performed from extracted DNA of each sample using barcoded PCR forward (5'- TCGTCGGCAGCGTCAGATGTGTATAAGAGACAGCCTACGGGNGGCWGCAG-3') and reverse (5'-GTCTCGTGGGCTCGGAGATGTGTATAAGAGACAGGACTACHVGGGTATCTAATCC-3') universal primers [57] containing the A and B adaptor sequences targeting the hypervariable V3-V4 region of the 16S rRNA gene. PCR amplicon products from all samples were purified using Agencourt AMPure beads (Agencourt, USA), and sequencing was performed on an Illumina MiSeq platform (NICEM, Republic of Korea). The raw fastq files were processed using the ‘quantitative insights into microbial ecology (QIIME)’ pipeline [58] Chimera and sequence reads < 200 bp and > 600 bp were removed. Gene sequences were separated from barcodes and primers. High-quality sequence reads were clustered into operational taxonomic units (OTUs) using a threshold of 97% pair-wise nucleotide sequence identity. OTUs were taxonomically classified using BLASTn against a curated GreenGenes database (May 2013 release), and using the Ribosomal Database Project (RDP) classifier (Sep 2016 release). Final data analysis was performed using OTUs assigned to specific taxonomic groups, excluding 47% OTUs not assigned to any taxonomic group. Relative abundance of OTUs at phylum level was compared among samples using the normalized OTU reads. To compare the bacterial community among samples, unconstrained principal coordination analysis (PCoA) was performed using the Bray-Curtis dissimilarity measures and plots were generated with R software (version 3.2.2) (http://www.r-project.org/) using Vegan and ggplot2 packages. Details regarding the ICS and AS samples, raw sequence data, and analyzed data are provided in SI (S8–S15 Tables).

### Accession numbers

Nucleotide accession numbers for the TCS resistance determinants of the TRG database were previously deposited [17] in the National Center for Biotechnology Information database and are included in tables and supplementary data where necessary. Moreover, information regarding FabI ENR substitutions associated TCS resistance were deposited to The Comprehensive Antibiotic Resistance Database (CARD) and can be accessed using the provided URL [59].

## Results and discussion

### TCS resistance determinants predicted by *in silico* confer TCS resistance

To confirm if the presence of potential TCS resistance gene can be used to predict TCS resistant phenotypes, we selected putative TCS resistance genes from WGS of five selected pathogenic bacteria and examined for contribution on TCS resistance in *E*. *coli*. WGS analysis of the selected 17 bacterial strains revealed the presence of various TCS resistance determinants (S1 Table) which might be associated with TCS resistance (Table 1). TCS resistance for the bacterial strains revealed that TCS resistant phenotype could be accurately predicted from the presence of putative TCS resistance genes, with high specificity-and sensitivity. Introduction of five selected TCS resistance determinants in an alternative host *E*. *coli* DH5α conferred expected levels of TCS resistance (S3 Table) with high specificity-and sensitivity. In our previous study, we successfully predicted bacterial TCS resistance based on the presence of putative TCS resistance gene [17], where genes encoding TCS tolerant metagenomic 7-α-HSDH in *Helicobacter pylori* and *Campylobacter jejuni* conferred significant levels of TCS resistance in a tested alternative host. WGS information of bacterial strains has been previously used to predict antimicrobial resistance profiles with high sensitivity and specificity [60–62]. Taken together, our results suggest that TRG database-based selection of TCS resistance is suitable to predict TCS resistance of bacterial pathogen. Other publicly available antibiotic resistant gene databases, either lack updated information about TCS resistance determinants or some of those information is redundant. For example searching various terms for “triclosan resistance” in the Antibiotic Resistance Genes Database (ARDB) [63] and The Comprehensive Antibiotic Resistance Database (CARD) [64] resulted in zero or single hits respectively. The BacMet database [56] on the other hand though contains many candidate TCS resistant gene homologues, however it has not been updated since January 18, 2014 and some of the genes such as *Acra*, *OprJ*, *OprN*, *TolC* among others, lack direct experimental evidence to confer TCS resistance individually.

### Majority of human and plant pathogens carry TCS resistance determinants

**Fig 1:**
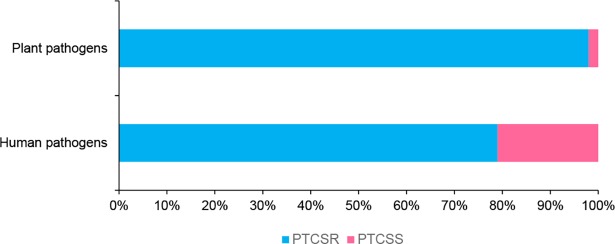
Triclosan resistance determinants were predominant in human-associated pathogenic bacteria and soil-borne plant pathogens. Organisms which carried or lack TCS resistance determinants were termed as potentially TCS resistant (PTCSR) or potentially TCS susceptible (PTCSS) respectively. Relative abundance of TCS resistance determinants in human-associated pathogenic bacteria and in soil-borne plant pathogenic bacteria; majority of the organism carried TCS resistance determinants.

*In silico* analysis of the genomes from 183 human-associated pathogenic/non-pathogenic and 48 soil-borne plant pathogenic bacteria revealed that the majority of these bacteria carried a variety of TCS resistance determinants (S4 and S5 Tables), Tables 2 and 3). Among the listed organisms, 78% of human-associated and 98% of soil-borne plant pathogens carried potential TCS resistance determinants in their genomes (Fig 1). These resistance determinants included completely TCS-tolerant ENR homologues such as FabV or 7-αHSDH, completely or moderately TCS resistant FabK, TCS-resistant FabI, or FabL, or *acrB* homologues. We found different combinations of these TCS resistance determinants, and furthermore, TCS resistant genes were either present as a single copy or co-occurred with other TCS resistance determinants. Based on the occurrence of these TCS resistance determinants in microorganisms, we identified certain resistance patterns (Tables 2 and 3). Organisms carrying either single or multiple copies of TCS tolerant ENRs such as FabV or 7-αHSDH homologues were categorized as being completely TCS refractory, whereas organisms with FabK ENR homologues were presumed either as completely TCS tolerant or moderately TCS resistant. Organisms carrying FabL, AcrB homologues, or FabI homologues carrying substitutions at key amino acid residues were considered TCS resistant at a specific concentration. Organisms solely possessing FabI without TCS resistance-associated substitutions were categorized as potentially susceptible. In fact, our *in silico* analysis found that 42% of human-associated pathogens and 52% of soil-borne plant pathogens had at least one TCS-tolerant ENR homologue (Tables 2 and 3), and that 57% of human-associated pathogens and 83% of soil-borne plant pathogens possessed multiple resistance determinants (Tables 2 and 3) that may confer complete tolerance to TCS. Bacteria with FabI ENR without any TCS resistance-associated substitutions were classified as potentially susceptible, and comprised a small proportion of bacterial pathogens (21% of human-associated pathogens and approximately 2% of soil-borne plant pathogens).

**Table 2:** Summary of TCS resistance determinants in 183 human-associated pathogenic and non-pathogenic bacteria.

TCS resistance determinants	Percent relative abundance	Expected phenotype
AcrB	58.4	Potentially resistant
FabL	9.8	Potentially mildly resistant
FabK	20.7	Potentially completely or moderately TCS tolerant
FabV	1.6	Potentially completely TCS tolerant
7-α-HSDH	23.4	Potentially completely TCS tolerant
FabI	100	Potential resistance in case of substitution at key enzyme sites
FabIG93A	42	Potentially resistant
FabIF203	24.5	Potentially resistant
FabI + AcrB	58.4	Potentially resistant
FabI + FabL	9.8	Potentially mildly resistant
FabI + FabV	1.6	Potentially completely TCS tolerant
FabI + FabK	20.7	Potentially completely or moderately TCS tolerant
FabI + 7-α HSDH	23.4	Potentially completely TCS tolerant
FabI + FabV + FabK + AcrB	0.5	Potentially completely TCS refractory
Organisms with two or more than two TCS resistance determinants	58.4	Potentially resistant
Organisms with at least one completely TCS resistance determinant homologue	42.6	Potentially completely TCS tolerant
Organisms with only FabI ENR homologue	33.3	Potential resistance in case of substitution at key enzyme sites
Organisms with only FabI ENR homologue which did not carry any substitution associated with TCS resistance	63	Potentially susceptible
Organisms with only FabI ENR homologue which carried substitution associated with TCS resistance	36	Potentially resistant
Among 183 FabI, which did not carry any TCS resistance associated substitutions	27.3	-
Among 183 FabI, which carried TCS resistance associated substitutions (Known, metagenomic, unknown)	72.6	Potentially resistant
Among 183 FabI, which carried TCS resistance associated substitutions (Known and metagenomic)	56.8	Potentially resistant
Potentially susceptible organisms among 183 human pathogens	21.3	1/4^th^ of the total organisms were potentially susceptible
Potentially resistant organisms among 183 human pathogens	78.6	3/4^th^ of the total organisms were potentially resistant

**Table 3:** Summary of TCS resistance determinants in 48 soil-borne plant pathogenic bacteria.

TCS resistance determinants	Percent relative abundance	Expected phenotype
AcrB	58.4	Potentially resistant
FabL	9.8	Potentially mildly resistant
FabK	20.7	Potentially completely or moderately TCS tolerant
FabV	1.6	Potentially completely TCS tolerant
7-α-HSDH	23.4	Potentially completely TCS tolerant
FabI	100	Potential resistance in case of substitution at key enzyme sites
FabIG93A	42	Potentially resistant
FabIF203	24.5	Potentially resistant
FabI + AcrB	58.4	Potentially resistant
FabI + FabL	9.8	Potentially mildly resistant
FabI + FabV	1.6	Potentially completely TCS tolerant
FabI + FabK	20.7	Potentially completely or moderately TCS tolerant
FabI + 7-α HSDH	23.4	Potentially completely TCS tolerant
FabI + FabV + FabK + AcrB	0.5	Potentially completely TCS refractory
Organisms with two or more than two TCS resistance determinants	58.4	Potentially resistant
Organisms with at least one completely TCS resistance determinant homologue	42.6	Potentially completely TCS tolerant
Organisms with only FabI ENR homologue	33.3	Potential resistance in case of substitution at key enzyme sites
Organisms with only FabI ENR homologue which did not carry any substitution associated with TCS resistance	63	Potentially susceptible
Organisms with only FabI ENR homologue which carried substitution associated with TCS resistance	36	Potentially resistant
Among 183 FabI, which did not carry any TCS resistance associated substitutions	27.3	-
Among 183 FabI, which carried TCS resistance associated substitutions (Known, metagenomic, unknown)	72.6	Potentially resistant
Among 183 FabI, which carried TCS resistance associated substitutions (Known and metagenomic)	56.8	Potentially resistant
Potentially susceptible organisms among 183 human pathogens	21.3	1/4^th^ of the total organisms were potentially susceptible
Potentially resistant organisms among 183 human pathogens	78.6	3/4^th^ of the total organisms were potentially resistant

### Mutations are predominant in FabI ENRs the single known effective target for TCS

TCS inhibits prototypic FabI ENR [54]; however, FabI is mutation-prone, and point mutation or combined mutations of the important catalytic residues in FabI ENRs confer TCS resistance [37–39]. Nevertheless, there is limited literature on TCS resistance-associated substitutions in FabI ENRs in other human pathogens and in the huge diversity of environmental microorganisms. Our in silico genome analysis revealed that most FabI ENRs from pathogenic organisms had specific amino acid substitutions, which may be associated with TCS resistance. Of those pathogenic microorganisms with FabI, we found that 56% of FabI ENRs in human-associated and approximately 69% of FabI ENRs in soil-borne plant pathogens had such substitutions (Tables 2 and 3, and S4 and S5 Tables). These substitutions were either present as point mutations or in combination. We found that the G93A substitution was abundant both in FabI ENRs from human-associated pathogens and from soil-borne plant pathogens. In addition to previously known substitutions, FabI ENRs from these organisms had novel substitutions, but whether these mutations confer resistance to TCS is not known. Substitutions at key amino acid residues in FabI ENR affect TCS binding efficacy in the active site pocket of ENR by changing conformation of the TCS binding site [65]. We hypothesize that the high structural diversity of FabI ENRs and various amino acid substitutions of the ENR are associated with TCS resistance, and will lead to different patterns of TCS resistance. This diversity and varied patterns of amino acids may alter the affinity of FabI ENRs to bind TCS, which may affect TCS activity in the organism. For instance, the diversity of amino acids constituting the TCS binding pocket may enhance or reduce binding of TCS to the ENR, and the subsequent resistance. Additionally, the frequent exposure of human-associated microorganisms and microorganisms in the environment to TCS may have already led to adaptations in these organisms by amino acid substitutions in FabI ENRs because of selective pressure.

### FabI is not always present as a single target ENR and is frequently co-localized with mild or completely TCS-resistant ENRs or AcrB efflux pumps

Another major concern associated with FabI-mediated TCS resistance is that FabI is not always present as a single ENR in a number of microorganisms [32]. Our *in silico* study reveals that only 33% of human-associated pathogens and approximately 2% of soil-borne plant pathogens carried FabI as a single target, and most of those FabI orthologues carried TCS resistance-associated substitutions (Tables 2 and 3, S4 and S5 Tables). Many of the microorganisms carry FabI ENR in combination with either mildly or completely TCS- resistant ENR homologues. Co-localization with mildly TCS-resistant ENRs might confer moderate resistance to this biocide, whereas FabI co-localized with TCS-refractory ENRs may render the organism fully resistant to TCS. Our results indicate that the majority of microorganisms have various combinations of ENRs in their genome. We found a predominant (23%) co-occurrence of FabI ENR with 7-αHSDH ENR in human-associated pathogenic organisms (Table 2). Other TCS-refractory/TCS-resistant ENRs that co-localized with FabI in human-associated pathogens include FabK (20%), FabV (1.6%), and FabL (9%) (Table 2). In regards to soil-borne plant pathogenic bacteria, we found that the AcrB efflux pump was predominantly (52%) co-localized with FabI, while FabV (approximately 8%) and FabK (approximately 11%) also occurred with FabI ENRs (Table 3). Because our findings indicated the presence of multiple TCS-resistant determinants in a number of single microbial genomes, we propose that the use of FabI inhibitors or TCS against such microorganisms may not be effective because of the presence of additional ENRs in their genomes. In fact, previous studies identified organisms with FabI that had mild or completely TCS-resistant ENRs, or AcrB efflux pumps such as *Pseudomonas aeruginosa* (FabI and FabV) [66–67], *Bacillus subtilis* (FabI and FabL) [68], and *Enterococcus faecalis* (FabI and FabK) [32]. These organisms exhibited significantly increased TCS resistance because of the presence of additional TCS tolerant or resistant ENR homologues.

### *In silico* analysis may accurately predict TCS tolerant superbugs

Our *in silico* genome comparisons revealed that completely TCS-tolerant ENRs were predominant in most examined pathogens both in human-associated (42%) and soil-borne plant pathogens (52%) (S4 and S5 Tables, S16 and S17 Tables). We found that the majority of human-associated bacteria that carried TCS-tolerant ENRs were pathogens (S4 Table, S16 Table), and that some had multiple TCS resistance determinants in the genome. These pathogenic bacteria were well-known human pathogens that cause various infections such as enteric diseases, opportunistic infections, skin and nosocomial infections, and gastric ulcers. Similarly, most of the plant pathogenic bacteria, which cause diseases in a variety of plants, carried TCS-tolerant ENRs in their genomes, such as FabV and FabK ENR homologues (S5 and S17 Tables). We propose that those organisms with TCS-tolerant ENR homologues likely confer resistance against TCS similar to that in previously identified organisms that have completely TCS-resistant ENRs [31–32, 66–67].

### Co-localized AcrB with FabI or with other ENRs is predominant in the genomes of most organisms—a potential determinant for co- and cross-resistance

The homotrimer AcrB, which acts as a tripartite complex, is the principal multidrug transporter in Gram-negative bacteria and confers antibiotic drug tolerance [68]. Our *in silico* analysis revealed that genes encoding the AcrB efflux pump were present in the majority of the human pathogenic bacteria (58.4%) and plant pathogens (87.5%) examined in this study (Tables 2 and 3). Moreover, AcrB homologues in these organisms were mostly found to be co-localized with other TCS resistance determinants such as FabI, FabV, FabL, 7-α HSDH, or FabK ENR homologues. The AcrB efflux subunit confers resistance against TCS [11, 17] in addition to co- and cross-resistance against other antibiotics [8, 14]. Biocides are known to potentially co-select for antibiotic resistance in bacteria [69], therefore, the excessive use of TCS may selectively enrich those organisms that have intrinsic determinants for TCS and other types of antibiotic resistance.

### Microbiome analysis revealed the presence of bacterial genera with potentially TCS tolerant pathogenic organisms

**Fig 2:**
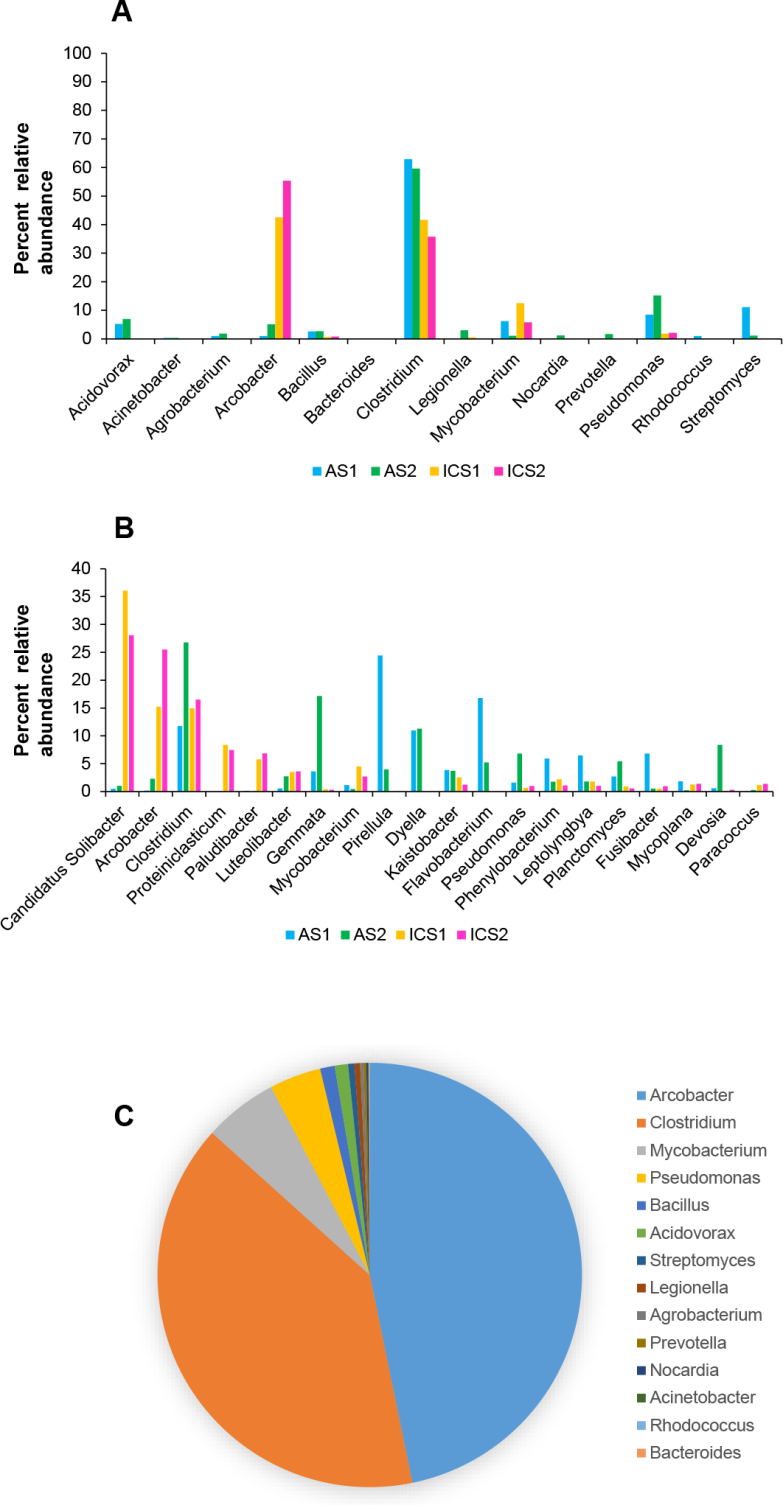
Bacterial genera with potentially TCS tolerant pathogenic organisms were present in TCS contaminated environments. **(A)** Percent relative abundance of candidate genera from AS and ICS with potentially pathogenic microorganisms. *Clostridium*, *Arcobacter*, *Mycobacterium*, and *Pseudomonas* were the major genera among the potentially pathogenic genera. **(B)** Percent relative abundance of the top 20 major genera from AS and ICS. Major genera include *Candidatus* Solibacter with metagenomic FabG and *Clostridium* with metagenomic 7AHSDH-like ENR homologues. Other genera with potentially pathogenic candidates in the top 20% genera included *Mycobacterium* and *Pseudomonas***. (C)** Cumulative relative abundance of genera with potentially pathogenic candidates from AS and ICS. Genera such as *Arcobacter*, *Clostridium*, *Mycobacterium*, *Pseudomonas*, *Bacillus*, and *Acidovorax* represented the major genera.

Microbiome analysis of AS and ICS revealed that bacterial genera with potentially pathogenic candidates were present and carried TRG homologues (Fig 2A, S13 Table). Those genera with potentially pathogenic candidates include *Clostridium*, *Arcobacter*, *Mycobacterium*, and *Pseudomonas*. Further, microbial community structure displayed similarity within and difference among AS and ICS samples, based on Bray-Curtis dissimilarity measures visualized by PCoA and comparison of relative abundance of bacterial taxa at phylum level (S1 Fig). Microbial community of two ICS samples were highly similar while that of two AS samples were quite dissimilar each other. This suggests that AS from the river estuarine may have diverse microbial community dependent on the location. However, our analysis with only two samples per site has a limitation to make a decisive conclusion on microbial community structure, which will be a subject of further study. Analysis of the relative abundance of the representative genera showed that *Arcobacter* (ranked 2^nd^), *Clostridium* (ranked 3^rd^), *Mycobacterium* (ranked 8^th^), and *Pseudomonas* (ranked 12^th^) were among the top 20 abundant genera (Fig 2B, S14 Table). Cumulative relative abundance analysis of those genera with potentially pathogenic candidates revealed that *Arcobacter*, *Clostridium*, *Mycobacterium*, *Pseudomonas*, *Bacillus*, and *Acidovorax* were the major genera (Fig 2C, S15 Table). *In silico* analysis showed that selected pathogenic bacterial strains from these genera had various potential TCS resistance determinants (S18 Table), and furthermore, it was found that these representative pathogenic genera carry potentially TCS-resistant determinants [17]. Previous studies have found that highly abundant genera such as *Candidatus* Solibacter, *Clostridium*, and *Pseudomonas* have completely TCS-tolerant ENR homologues [17, 67]. We propose that organisms with intrinsic TCS-tolerant determinants have additional benefits to flourish and be selectively enriched in TCS contaminated environments. However, it will be interesting to investigate how the population of TCS resistant pathogenic and non-pathogenic bacteria will change over time in a diverse microbial community under TCS selective pressure.

## Conclusions

We conclude that TCS resistance determinants are highly abundant in most human pathogenic bacteria and in the majority of plant pathogenic bacteria, and that TCS may not be as effective against those organisms as previously presumed. Since FabI is targeted by other clinically important antimicrobials, and most organisms possess intrinsic TCS tolerance determinants, the continuously escalating use of this biocide may not only exert a selective pressure for TCS resistance, but also enrich for other antibiotic resistance genes in the environment. Furthermore, co-localization of a diverse number of TCS resistant ENRs with FabI may render TCS and TCS-based ENR inhibitors ineffective as antimicrobial agents. Therefore, it is important that the diversity of ENRs in pathogenic bacteria should be considered prior to developing selective ENR inhibitors.

## Supporting information

S1 Fig Community structure displayed similarity within and difference among AS and ICS samples. (a) Principal coordinate analysis (PCoA) plot representing differences in microbial community among AS and ICS samples. Each point represents individual sample. The variance explained by the PCoA is indicated on the axes. (b) Percent relative abundance revealed relatively similar microbial community structure among similar sample types.
(TIF)

S1 Table Details of the genome wide analysis of selected 17 bacterial strains to predict phenotype from genotype in terms of the presence/absence of TCS resistance determinants. (XLSX)

S2 Table List of primers used in PCR reaction for predicted TCS resistance determinants to clone into pGEM-T Easy. (XLSX)

S3 Table In vivo TCS resistance patterns of representative TRG homologues from tested strains cloned into pGEM-T Easy in E. coli DH5α. (XLSX)

S4 Table Details of the genome wide analysis of the human pathogens for TCS resistance determinants. (XLSX)

S5 Table Details of the genome wide analysis of the plant pathogens for TCS resistance determinants. (XLSX)

S6 Table Details of the constructed TRG database used for genome wide analysis of the pathogenic bacteria. (XLSX)

S7 Table Rationale for selecting AcrB as a representative of TCS resistant (TCSR) efflux pump protein homologue. (XLSX)

S8 Table Miseq sequence details of the TCS contaminated soil samples, used for microbiome analysis. (XLSX)

S9 Table Candidate genera from ICS and AS samples determined by Miseq sequence analysis (OTU count). (XLSX)

S10 Table Bacterial genera that carried human and plant pathogenic bacteria and were present in Elsukdo AS and Sasang ICS are highlighted in bold dark red. (XLSX)

S11 Table Relative abundance of bacterial genera in AS and ICS. (XLSX)

S12 Table Relative abundance of bacterial genera in AS and ICS. (XLSX)

S13 Table This data was used for preparing Fig 2A. (XLSX)

S14 Table This data was used for preparing Fig 2B. (XLSX)

S15 Table This data was used for preparing Fig 2C. (XLSX)

S16 Table Human pathogenic and non-pathogenic bacteria with completely TCS tolerant ENR homologues. (XLSX)

S17 Table Soil-borne plant pathogenic bacteria with completely TCS tolerant ENR. (XLSX)

S18 Table Potential TCS resistant determinants in bacterial genera with pathogenic candidates. (XLSX)

## References

[1] Wellington EM, Boxall AB, Cross P, Feil EJ, Gaze WH, Hawkey PM, et al The role of the natural environment in the emergence of antibiotic resistance in Gram-negative bacteria. Lancet Infect Dis. 2013;13: 155–65. doi: 10.1016/S1473-3099(12)70317-1 23347633 10.1016/S1473-3099(12)70317-1

[2] Walsh F. Investigating antibiotic resistance in non-clinical environments. Front Microbiol. 2013;4: 19 doi: 10.3389/fmicb.2013.00019 23423602 10.3389/fmicb.2013.00019 PMC3573686

[3] Fraise AP. Biocide abuse and antimicrobial resistance—a cause for concern?. J Antimicrob Chemother. 2002;49: 11–2. 10.1093/jac/49.1.11 11751760

[4] Halden RU. On the need and speed of regulating triclosan and triclocarban in the United States. Environ Sci Technol. 2014;48: 3603–11. doi: 10.1021/es500495p 24588513 10.1021/es500495p PMC3974611

[5] Yueh MF, Tukey RH. Triclosan: a widespread environmental toxicant with many biological effects. Annu Rev Pharmacol Toxicol. 2016; 56:251–72. doi: 10.1146/annurev-pharmtox-010715-103417 26738475 10.1146/annurev-pharmtox-010715-103417 PMC4774862

[6] Saleh S, Haddadin RN, Baillie S, Collier PJ. Triclosan–an update. Lett Appl Microbiol. 2011;52: 87–95. doi: 10.1111/j.1472-765X.2010.02976.x 21166831 10.1111/j.1472-765X.2010.02976.x

[7] Heath RJ, Rubin JR, Holland DR, Zhang E, Snow ME, Rock CO. Mechanism of triclosan inhibition of bacterial fatty acid synthesis. J Biol Chem. 1999;274: 11110–4. 10196195 10.1074/jbc.274.16.11110

[8] Yazdankhah SP, Scheie AA, Høiby EA, Lunestad BT, Heir E, Fotland TØ, et al Triclosan and antimicrobial resistance in bacteria: an overview. Microb Drug Resist. 2006;12: 83–90. doi: 10.1089/mdr.2006.12.83 16922622 10.1089/mdr.2006.12.83

[9] McMurry LM, McDermott PF, Levy SB. Genetic evidence that InhA of *Mycobacterium smegmatis* is a target for triclosan. Antimicrob Agents Chemother. 1999;43: 711–3. 10049298 10.1128/aac.43.3.711 PMC89191

[10] Russell AD. Whither triclosan?. J Antimicrob Chemother. 2004;53: 693–5. doi: 10.1093/jac/dkh171 15073159 10.1093/jac/dkh171

[11] Mcmurry LM, Oethinger M, Levy SB. Overexpression of *marA*, *soxS*, or *acrAB* produces resistance to triclosan in laboratory and clinical strains of *Escherichia coli*. FEMS Microbiol Lett. 1998;166: 305–9. 9770288 10.1111/j.1574-6968.1998.tb13905.x

[12] Fernando DM, Xu W, Loewen PC, Zhane GG, Kumar A. Triclosan can select for an AdeIJK-overexpressing mutant of *Acinetobacter baumannii* ATCC 17978 that displays reduced susceptibility to multiple antibiotics. Antimicrob Agents Chemother. 2014;58: 6424–31. doi: 10.1128/AAC.03074-14 25136007 10.1128/AAC.03074-14 PMC4249441

[13] Schweizer HP. Triclosan: a widely used biocide and its link to antibiotics. FEMS Microbiol Lett. 2001; 202: 1–7. 11506900 10.1111/j.1574-6968.2001.tb10772.x

[14] Chuanchuen R, Beinlich K, Hoang TT, Becher A, Karkhoff-Schweizer RR, Schweizer HP. Cross-Resistance between triclosan and antibiotics in *Pseudomonas aeruginosa* is mediated by multidrug efflux pumps: exposure of a susceptible mutant strain to triclosan selects *nfxB* mutants overexpressing MexCD-OprJ. Antimicrob Agents Chemother. 2001;45: 428–32. doi: 10.1128/AAC.45.2.428-432.2001 11158736 10.1128/AAC.45.2.428-432.2001 PMC90308

[15] Bailey AM, Paulsen IT, Piddock LJ. RamA confers multidrug resistance in *Salmonella enterica* via increased expression of acrB, which is inhibited by chlorpromazine. Antimicrob Agents Chemother. 2008;52: 3604–11 doi: 10.1128/AAC.00661-08 18694955 10.1128/AAC.00661-08 PMC2565896

[16] Yu BJ, Kim JA, Pan JG. Signature gene expression profile of triclosan-resistant *Escherichia coli*. J Antimicrob Chemother. 2010;65: 1171–7. doi: 10.1093/jac/dkq114 20410062 10.1093/jac/dkq114

[17] Khan R, Kong HG, Jung YH, Choi J, Baek KY, Hwang EC, et al Triclosan resistome from metagenome reveals diverse enoyl acyl carrier protein reductases and selective enrichment of triclosan resistance genes. Sci Rep. 2016;6: 32322 doi: 10.1038/srep32322 27577999 10.1038/srep32322 PMC5006077

[18] FDA Issues Final Rule on Safety and Effectiveness of Antibacterial Soaps. 2016. [Last retrieved on 2018 Jan 10]. Available from: http://www.fda.gov/NewsEvents/Newsroom/PressAnnouncements/ucm517478.htm.

[19] Macri D. Worldwide use of triclosan: Can dentistry do without this antimicrobial?. Contemp Clin Dent. 2017; 8: 7 doi: 10.4103/ccd.ccd_225_17 28566843 10.4103/ccd.ccd_225_17 PMC5426170

[20] Dinwiddie MT, Terry PD, Chen J. Recent evidence regarding triclosan and cancer risk. Int J Environ Res Public Health. 2014;11: 2209–17. doi: 10.3390/ijerph110202209 24566048 10.3390/ijerph110202209 PMC3945593

[21] James M, Ambadapadi S, Falany C. Triclosan inhibits the activity of expressed human sulfotransferases (SULTs) towards their diagnostic substrates. FASEB J. 2015;29(1 Supplement): 622–4.

[22] Sippel KH, Vyas NK, Zhang W, Sankaran B, Quiocho FA. Crystal structure of the human fatty acid synthase enoyl-acyl carrier protein-reductase domain complexed with triclosan reveals allosteric protein-protein interface inhibition. J. Biol. Chem. 2014;289: 33287–95. doi: 10.1074/jbc.M114.608547 25301948 10.1074/jbc.M114.608547 PMC4246086

[23] Marshall NB, Lukomska E, Long CM, Kashon ML, Sharpnack DD, Nayak AP, Anderson KL, Jean Meade B, Anderson SE. Triclosan induces thymic stromal lymphopoietin in skin promoting Th2 allergic responses. Toxicol. Sci. 2015;147: 127–39. doi: 10.1093/toxsci/kfv113 26048654 10.1093/toxsci/kfv113 PMC4734116

[24] Marshall N, Long C, Anderson K, Lukomska E, Anderson S. Dermal exposure to the commonly used antimicrobial chemical triclosan promotes allergic responses in skin and lung (HYP7P. 317). J. Immunol. 2014;192(1 Supplement): 119–32.

[25] Walters GI, Robertson AS, Moore VC, Burge PS. Occupational asthma caused by sensitization to a cleaning product containing triclosan. Ann. Allergy Asthma Immunol. 2017;118: 370–1. doi: 10.1016/j.anai.2016.12.001 28065800 10.1016/j.anai.2016.12.001

[26] Yee AL, Gilbert JA. Is triclosan harming your microbiome?. Science. 2016;353: 348–9. doi: 10.1126/science.aag2698 27463658 10.1126/science.aag2698

[27] Gao B, Tu P, Bian X, Chi L, Ru H, Lu K. Profound perturbation induced by triclosan exposure in mouse gut microbiome: a less resilient microbial community with elevated antibiotic and metal resistomes. BMC Pharmacol Toxicol. 2017;18: 46 doi: 10.1186/s40360-017-0150-9 28606169 10.1186/s40360-017-0150-9 PMC5469155

[28] Jackowski S, Murphy CM, Cronan JE, Rock CO. Acetoacetyl-acyl carrier protein synthase. A target for the antibiotic thiolactomycin. J Biol Chem. 1989;264: 7624–9. 2651445

[29] Bergler H, Wallner P, Ebeling A, Leitinger B, Fuchsbichler S, Aschauer H, et al Protein EnvM is the NADH-dependent enoyl-ACP reductase (FabI) of *Escherichia coli*. J Biol Chem. 1994;269: 5493–6. 8119879

[30] Heath RJ, Su N, Murphy CK, Rock CO. The enoyl-[acyl-carrier-protein] reductases FabI and FabL from *Bacillus subtilis*. J Biol Chem. 2000;275: 40128–33. doi: 10.1074/jbc.M005611200 11007778 10.1074/jbc.M005611200

[31] Massengo-Tiassé RP, Cronan JE. *Vibrio cholerae* FabV defines a new class of enoyl-acyl carrier protein reductase. J Biol Chem. 2008;283: 1308–16. doi: 10.1074/jbc.M708171200 18032386 10.1074/jbc.M708171200

[32] Heath RJ, Rock CO. Microbiology: A triclosan-resistant bacterial enzyme. Nature. 2000;406: 145–6. doi: 10.1038/35018162 10910344 10.1038/35018162

[33] White SW, Zheng J, Zhang YM, Rock CO. The structural biology of type II fatty acid biosynthesis. Annu Rev Biochem. 2005;74: 791–831. doi: 10.1146/annurev.biochem.74.082803.133524 15952903 10.1146/annurev.biochem.74.082803.133524

[34] Joernvall H, Persson B, Krook M, Atrian S, Gonzalez-Duarte R, Jeffery J, et al Short-chain dehydrogenases/reductases (SDR). Biochemistry. 1995;34: 6003–6013. 7742302 10.1021/bi00018a001

[35] Kallberg Y, Oppermann U, Jörnvall H, Persson B. Short‐chain dehydrogenases/reductases (SDRs). Eur J Biochem. 2002;269: 4409–17. 12230552 10.1046/j.1432-1033.2002.03130.x

[36] Persson B, Kallberg Y, Oppermann U, Jörnvall H. Coenzyme-based functional assignments of short-chain dehydrogenases/reductases (SDRs). Chem Biol Interact. 2003;143: 271–78. 12604213 10.1016/s0009-2797(02)00223-5

[37] McMurry LM, Oethinger M, Levy SB. Triclosan targets lipid synthesis. Nature. 1998;394: 531–2. doi: 10.1038/28970 9707111 10.1038/28970

[38] Fan F, Yan K, Wallis NG, Reed S, Moore TD, Rittenhouse SF, et al Defining and combating the mechanisms of triclosan resistance in clinical isolates of *Staphylococcus aureus*. Antimicrob Agents Chemother. 2002;46: 3343–7 doi: 10.1128/AAC.46.11.3343-3347.2002 12384334 10.1128/AAC.46.11.3343-3347.2002 PMC128739

[39] Stewart MJ, Parikh S, Xiao G, Tonge PJ, Kisker C. Structural basis and mechanism of enoyl reductase inhibition by triclosan. J Mol Biol. 1999;290: 859–65. doi: 10.1006/jmbi.1999.2907 10398587 10.1006/jmbi.1999.2907

[40] Campbell JW, Cronan Jr JE. Bacterial fatty acid biosynthesis: targets for antibacterial drug discovery. Annu Rev Microbiol. 2001;55: 305–32. doi: 10.1146/annurev.micro.55.1.305 11544358 10.1146/annurev.micro.55.1.305

[41] Biswas S, Raoult D, Rolain JM. A bioinformatic approach to understanding antibiotic resistance in intracellular bacteria through whole genome analysis. Int J Antimicrob Agents. 2008;32: 207–20. doi: 10.1016/j.ijantimicag.2008.03.017 18619818 10.1016/j.ijantimicag.2008.03.017

[42] Poirel L, Bonnin RA, Nordmann P. Analysis of the resistome of a multidrug-resistant NDM-1-producing *Escherichia coli* strain by high-throughput genome sequencing. Antimicrob Agents Chemother. 2011;55: 4224–29. doi: 10.1128/AAC.00165-11 21746951 10.1128/AAC.00165-11 PMC3165296

[43] Zankari E, Hasman H, Cosentino S, Vestergaard M, Rasmussen S, Lund O, et al Identification of acquired antimicrobial resistance genes. J Antimicrob Chemother. 2012;67: 2640–44. doi: 10.1093/jac/dks261 22782487 10.1093/jac/dks261 PMC3468078

[44] Rout S, Warhurst DC, Suar M, Mahapatra RK. *In silico* comparative genomics analysis of *Plasmodium falciparum* for the identification of putative essential genes and therapeutic candidates. J Microbiol Methods. 2015;109: 1–8. doi: 10.1016/j.mimet.2014.11.016 25486552 10.1016/j.mimet.2014.11.016

[45] Chong CE, Lim BS, Nathan S, Mohamed R. *In silico* analysis of *Burkholderia pseudomallei* genome sequence for potential drug targets. In silico Biol. 2006;6: 341–6. 16922696

[46] Forsberg KJ, Patel S, Gibson MK, Lauber CL, Knight R, Fierer N, et al Bacterial phylogeny structures soil resistomes across habitats. Nature. 2014;509: 612–6. doi: 10.1038/nature13377 24847883 10.1038/nature13377 PMC4079543

[47] Sambrook J, Fritsch E, Maniatis T. Molecular Cloning: a Laboratory Manual. 4th ed New York: Cold Spring Harbor Laboratory Press; 1989.

[48] Basic Local Alignment Search Tool. Available from: http://blast.ncbi.nlm.nih.gov.

[49] Bester K. Triclosan in a sewage treatment process—balances and monitoring data. Water Res. 2003;37: 3891–6. doi: 10.1016/S0043-1354(03)00335-X 12909107 10.1016/S0043-1354(03)00335-X

[50] Butler E, Whelan MJ, Sakrabani R, Van Egmond R. Fate of triclosan in field soils receiving sewage sludge. Environ. Pollut. 2012;167: 101–9. doi: 10.1016/j.envpol.2012.03.036 22561896 10.1016/j.envpol.2012.03.036

[51] Singer H, Müller S, Tixier C, Pillonel L. Triclosan: occurrence and fate of a widely used biocide in the aquatic environment: field measurements in wastewater treatment plants, surface waters, and lake sediments. Environ Sci Technol. 2002;36: 4998–5004. 12523412 10.1021/es025750i

[52] Parkpian P, Tet Leong S, Laortanakul P, Thi Kim Phuong N. The benefits and risks of using river sediment for Vietnamese agriculture: a case study of the Nhieu Loc canal in Ho Chi Minh city. J Environ Sci Health A Tox Hazard Subst Environ Eng. 2002;37: 1099–1122. 10.1081/ESE-120004526 28880803

[53] Supplementary Data 2, sheet 2 named as TRG-Reference database. Available from: http://www.nature.com/article-assets/npg/srep/2016/160831/srep32322/extref/srep32322-s3.xls

[54] Levy CW, Roujeinikova A, Sedelnikova S, Baker PJ, Stuitje AR, Slabas AR, et al Molecular basis of triclosan activity. Nature. 1999;398: 383–4. doi: 10.1038/18803 10201369 10.1038/18803

[55] Poole K. Efflux-mediated antimicrobial resistance. J Antimicrob Chemother. 2005;56: 20–51. doi: 10.1093/jac/dki171 15914491 10.1093/jac/dki171

[56] Pal C, Bengtsson-Palme J, Rensing C, Kristiansson E, Larsson DGJ. BacMet: antibacterial biocide and metal resistance genes database. Nucleic Acid Res. 2014;42: (D1),D737–D743. 24304895 10.1093/nar/gkt1252 PMC3965030

[57] Klindworth A, Pruesse E, Schweer T, Peplies J, Quast C, Horn M, Glöckner FO. Evaluation of general 16S ribosomal RNA gene PCR primers for classical and next-generation sequencing-based diversity studies. Nucleic Acids Res. 2013;41: e1 doi: 10.1093/nar/gks808 22933715 10.1093/nar/gks808 PMC3592464

[58] Caporaso JG, Kuczynsk J, Stombaugh J, Bittinger K, Bushman FD, Costello EK, et al QIIME allows analysis of high-throughput community sequencing data. Nat Methods. 2010;7: 335–6. doi: 10.1038/nmeth.f.303 20383131 10.1038/nmeth.f.303 PMC3156573

[59] The Comprehensive Antibiotic Resistance Database. Available from: https://card.mcmaster.ca/ontology/41097.

[60] Tyson GH, McDermott PF, Li C, Chen Y, Tadesse DA, Mukherjee S, et al WGS accurately predicts antimicrobial resistance in *Escherichia coli*. J Antimicrob Chemother. 2015;70: 2763–9. doi: 10.1093/jac/dkv186 26142410 10.1093/jac/dkv186 PMC11606221

[61] Zhao S, Tyson GH, Chen Y, Li C, Mukherjee S, Young S, et al Whole-genome sequencing analysis accurately predicts antimicrobial resistance phenotypes in *Campylobacter* spp. Appl Environ Microbiol. 2016;82: 459–66. 10.1128/AEM.02873-15 PMC4711122 26519386

[62] McDermott P, Tyson GH, Kabera C, Chen Y, Li C, Folster JP, et al Whole-genome sequencing for detecting antimicrobial resistance in nontyphoidal *Salmonella*. Antimicrob Agents Chemother. 2016;60: 5515–20. doi: 10.1128/AAC.01030-16 27381390 10.1128/AAC.01030-16 PMC4997858

[63] Liu B, Pop M. ARDB—antibiotic resistance genes database. Nucleic Acids Res. 2009;37: D443–D447. doi: 10.1093/nar/gkn656 18832362 10.1093/nar/gkn656 PMC2686595

[64] Jia B, Raphenya AR, Alcock B, Waglechner N, Guo P, Tsang KK, et al CARD 2017: expansion and model-centric curation of the comprehensive antibiotic resistance database. Nucleic Acids Res. 2017;45: D566–D573. doi: 10.1093/nar/gkw1004 27789705 10.1093/nar/gkw1004 PMC5210516

[65] Stewart MJ, Parikh S, Xiao G, Tonge PJ, Kisker C. Structural basis and mechanism of enoyl reductase inhibition by triclosan. J Mol Biol. 1999;290: 859–65. doi: 10.1006/jmbi.1999.2907 10398587 10.1006/jmbi.1999.2907

[66] Hoang TT, Schweizer HP. Characterization of *Pseudomonas aeruginosa* enoyl-acyl carrier protein reductase (FabI): a target for the antimicrobial triclosan and its role in acylated homoserine lactone synthesis. J Bacteriol. 1999;181: 5489–97. 10464225 10.1128/jb.181.17.5489-5497.1999 PMC94060

[67] Zhu L, Lin J, Ma J, Cronan JE, Wang H. Triclosan resistance of *Pseudomonas aeruginosa* PAO1 is due to FabV, a triclosan-resistant enoyl-acyl carrier protein reductase. Antimicrob Agents Chemother. 2010;54: 689–98. doi: 10.1128/AAC.01152-09 19933806 10.1128/AAC.01152-09 PMC2812149

[68] Nakashima R, Sakurai K, Yamasaki S, Nishino K, Yamaguchi A. Structures of the multidrug exporter AcrB reveal a proximal multisite drug-binding pocket. Nature. 2011;480: 565–69. doi: 10.1038/nature10641 22121023 10.1038/nature10641

[69] Pal C, Bengtsson-Palme J, Kristiansson E, Larsson DJ. Co-occurrence of resistance genes to antibiotics, biocides and metals reveals novel insights into their co-selection potential. BMC genomics. 2015;16: 964 doi: 10.1186/s12864-015-2153-5 26576951 10.1186/s12864-015-2153-5 PMC4650350

