# Supplementary figures and images for: Distribution of triclosan-resistant genes in major pathogenic microorganisms revealed by metagenome and genome-wide analysis
Source: PLoS One. 2018 Feb 8;13(2):e0192277. doi: 10.1371/journal.pone.0192277 (PMC5805296; doi:10.1371/journal.pone.0192277)

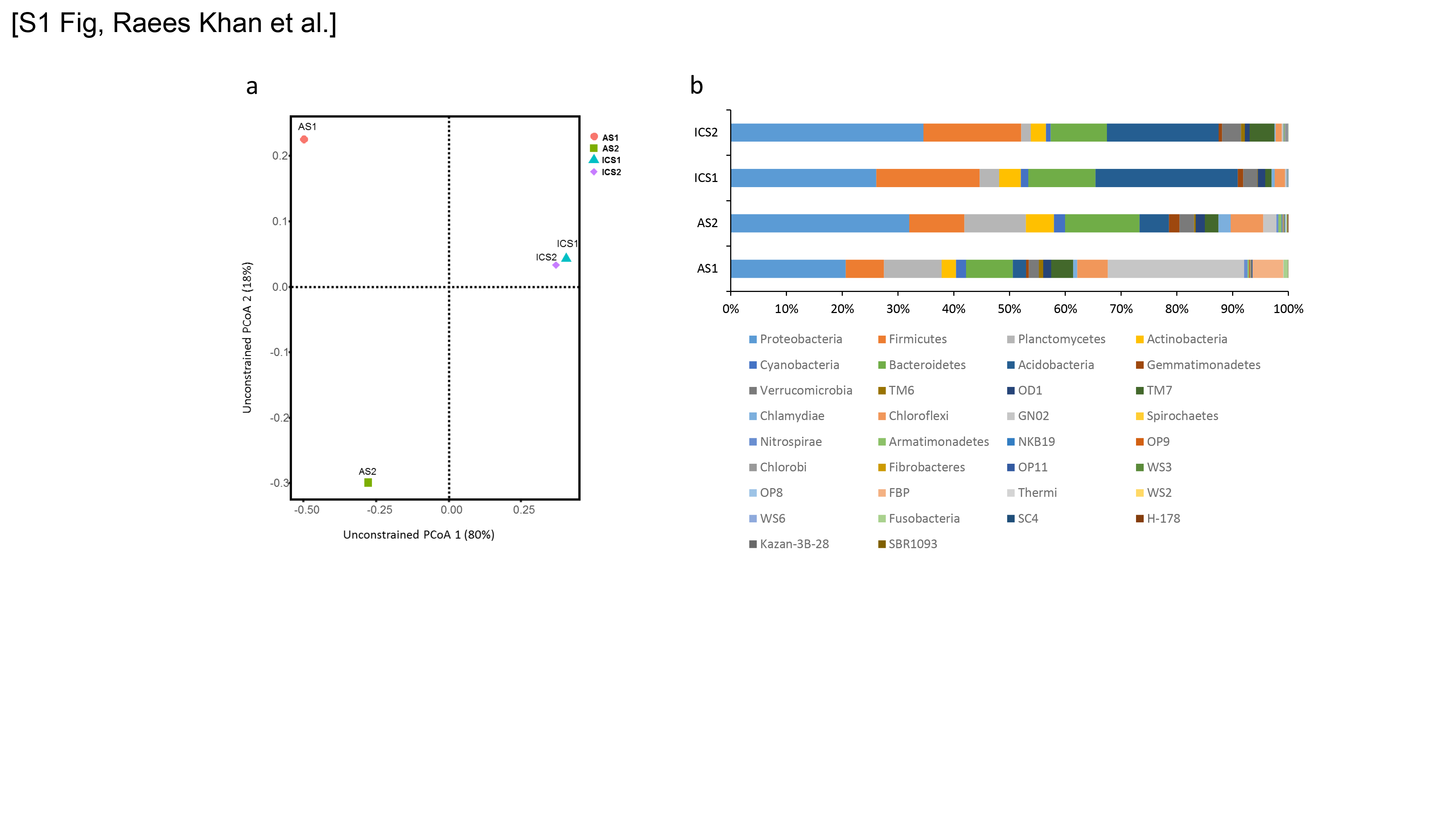

Supplement: S1 Fig — (a) Principal coordinate analysis (PCoA) plot representing differences in microbial community among AS and ICS samples. Each point represents individual sample. The variance explained by the PCoA is indicated on the axes. (b) Percent relative abundance revealed relatively similar microbial community structure among similar sample types. (TIF) [file pone.0192277.s001.tif]
